# Redox mediators towards practical aqueous batteries

**DOI:** 10.1093/nsr/nwae320

**Published:** 2024-09-10

**Authors:** Jiafeng Lei, Yi-Chun Lu

**Affiliations:** Electrochemical Energy and Interfaces Laboratory, Department of Mechanical and Automation Engineering, The Chinese University of Hong Kong, China; Electrochemical Energy and Interfaces Laboratory, Department of Mechanical and Automation Engineering, The Chinese University of Hong Kong, China

**Figure 1.**
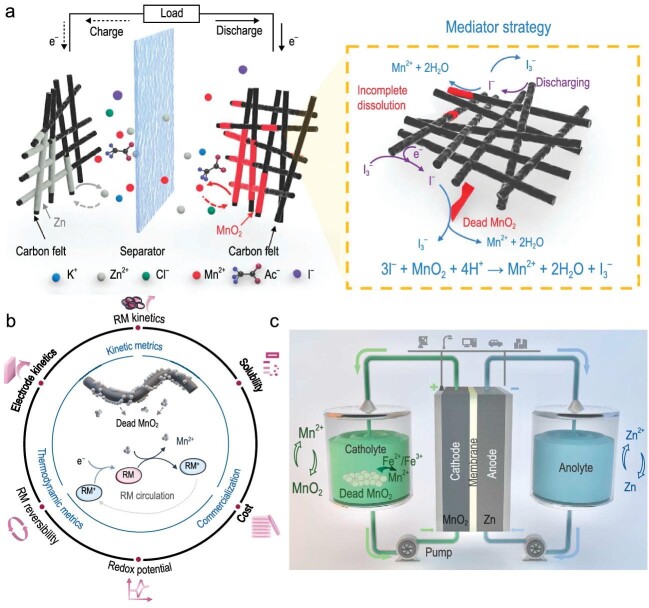
(a) Schematic illustration of the mediator effect in zinc manganese batteries with iodide as the RM to promote MnO_2_ dissolution. Adapted with permission from ref. [3]. (b) Critical factors of mediators that control Zn-Mn electrolytic cell performance. (c) Schematic illustration of the Zn-Mn electrolytic flow cell with Fe^2+^/Fe^3+^ as the RM. Adapted with permission from ref. [5].

Aqueous Zn-Mn^2+^/MnO_2_ batteries with deposition-dissolution mechanisms have garnered much attention owing to their cost effectiveness, high voltage and high capacity (616 mAh g^–1^) [1]. For deposition-based batteries, areal capacity is one of the most important parameters determining the whole system's energy [2]. However, achieving high areal capacity is challenging for Mn^2+^/MnO_2_-based batteries because of the severe MnO_2_ exfoliations and incomplete dissolution, which is caused by the internal stress and poor conductivity of MnO_2_. Without contact with electrodes, ‘dead’ MnO_2_ leads to irreversible capacity loss and battery failure. Lu and co-workers first introduced iodide as the redox mediator (RM) to solve the ‘dead’ MnO_2_ issue (Fig. 1a) and unlock the areal capacity to 50 mAh cm^–2^ in the weak-acid acetate system (Equation (1)) [3]:


(1) \begin{eqnarray*}
&&3\,{{{\mathrm{I}}}^-} + {\mathrm{Mn}}{{{\mathrm{O}}}_2} + 4\,{{{\mathrm{H}}}^ + } \to {\mathrm{M}}{{{\mathrm{n}}}^{2 + }}\\
&&\quad +\, {{{\mathrm{I}}}_3}^- + 2\,{{{\mathrm{H}}}_2}{\mathrm{O}}
\end{eqnarray*}

Chen and the team used bromide as the RM in an acid sulfate system with a promoted output voltage of ∼2 V (Equation (2)) [4]. However, its effectiveness is hindered by the poor kinetics of bromide species:


(2) \begin{eqnarray*}
&&3\,{\mathrm{B}}{{{\mathrm{r}}}^-} + {\mathrm{Mn}}{{{\mathrm{O}}}_2} + 4\,{{{\mathrm{H}}}^ + } \to {\mathrm{M}}{{{\mathrm{n}}}^{2 + }}\\
&&\quad +\, {\mathrm{B}}{{{\mathrm{r}}}_3}^- + 2\,{{{\mathrm{H}}}_2}{\mathrm{O}}
\end{eqnarray*}

Exploring novel RMs with fast kinetics, suitable potential and high stability is crucial for practical high-energy Zn-Mn batteries. Recently, writing in *National Science Review*, Chao, Zhou and co-workers reported a bi-functional Fe^2+^/Fe^3+^ RM that could recover the irreversible capacity and catalyze MnO_2_ kinetics behaviors, which was a step forward for practical aqueous batteries [5].

RMs are electron shuttles that transfer electrons from electrodes to active materials via spontaneous chemical reactions, offering a feasible way to enhance the performance of aqueous batteries from various dimensions. In deposition-based systems, the diffusible RM could recover the irreversible capacity loss from the ‘dead’ active material (MnO_2_, Zn and other metals) that has lost contact with the electrode, and eliminate electrode/membrane passivation [3–5]. Furthermore, RMs may promote kinetics by transforming sluggish electrochemical processes into fast electrochemical and chemical processes [6]. The unique properties of an RM enable it to break the solubility limit by utilizing the capacity from solid active material in redox flow batteries through the ‘redox targeting strategy’ [7]. Moreover, an RM could stabilize electrode/electrolyte by alerting the reaction pathway [8]. It has been reported that RMs can manipulate morphology in Li-O_2_ and Li-S batteries to create 3D structures, shedding light on other aqueous battery systems with deposition-dissolution mechanisms [9].

Rational design and proper selection of RMs are essential (Fig. 1b). Potential match is the thermodynamic prerequisite of applying RMs in batteries, as the chemical reaction driving force comes from the potential difference between active materials and RMs (${\mathrm{\Delta }}$G $< $ 0). To facilitate MnO_2_ dissolution, the potential of an RM should be lower than that of the Mn^2+^/MnO_2_ reaction. With iron ions as RMs, after the active MnO_2_ is discharged, the Fe^3+^ ions first undergo electroreduction to Fe^2+^, followed by the chemical reaction with MnO_2_ and being oxidized to Fe^3+^ (Equation (3)). The oxidized Fe^3+^ ions then diffuse back to the electrode and repeat this cycle until all of the MnO_2_ is consumed:


(3) \begin{eqnarray*}
&&2\,{\mathrm{F}}{{{\mathrm{e}}}^{2 + }} + {\mathrm{Mn}}{{{\mathrm{O}}}_2} + 4\,{{{\mathrm{H}}}^ + } \to {\mathrm{M}}{{{\mathrm{n}}}^{2 + }}\\
&&\quad +\, 2\,{\mathrm{F}}{{{\mathrm{e}}}^{3 + }} + 2\,{{{\mathrm{H}}}_2}{\mathrm{O}}
\end{eqnarray*}

It should be noted that such a potential difference between an RM and MnO_2_ would inevitably result in energy loss, highlighting the trade-off between kinetics and energy efficiency.

A high electron transfer rate between RMs and active materials is crucial for achieving high utilization of active materials. The kinetics analysis indicates that Fe^2+^ has a high exchange current of 6.31 $\times $ 10^–11^ A, higher than the value of I^–^ (4.26 $\times $ 10^–12^ A) and Br^–^ (8 $\times $ 10^–14^ A). The fast kinetics is also supported by the *in-situ* ultraviolet-visible spectroscopy that Fe^2+^ ions rapidly react with MnO_2_ within a short time. With the assistance of the Fe^2+^/Fe^3+^ mediator, the aqueous Zn-Mn electrolytic flow cell (Fig. 1c) showed a high areal capacity of 80 mAh cm^–2^ (10% of the capacity contribution from the RM itself). Interestingly, iron ions are found to be doped in the electrodeposited Fe-MnO_2_ cathode with the generation of more oxygen vacancies, which greatly improves the conductivity and kinetics. Such electrochemical improvement aligns with previous studies applying transition metal (Ni, Co, etc.) doping strategies [10].

Future work on identifying the governing factors for RM kinetics (mediation reactions with active materials) is required for designing efficient RMs in practical application. It would be interesting to explore whether MnO_2_ crystal structure engineering facilitates mediation processes. Except for the electrode potential of RMs, the role of protons in MnO_2_ mediation reaction kinetics needs to be carefully evaluated because of their involvement in the mediation reaction (Equation (3)). The proton on the posolyte side will inevitably crossover to the negolyte side with severe hydrogen evolution reactions, while insufficient proton concentration may impair mediation kinetics, causing MnO_2_ accumulation. Constraining proton crossover is vital for long-term stability. Even though Fe^2+^/Fe^3+^ RMs hold great promise for Zn-Mn batteries, the voltage gap between Fe^2+^/Fe^3+^ and Mn^2+^/MnO_2_ (>500 mV) should be further reduced. At higher areal capacity (>100 mAh cm^–2^), most of the discharge capacity may result from the mediation reaction, where the low potential of RMs could lead to higher energy loss. Exploring novel RMs with high potential (ideally the potential difference between the RM and MnO_2_ is less than 200 mV), high stability and faster kinetics are highly desirable in this field.

In summary, Chao and colleagues reported a stable Fe^2+^-mediated Zn-Mn electrolytic flow cell. Not only do iron ions act as the RM to recover ‘lost’ capacity from ‘dead’ MnO_2_, but they also dope into the MnO_2_, catalyzing its electrolytic kinetics. This bi-functional mediator design provides a new path to practical applications of aqueous batteries.
